# The Association of *Mycobacterium avium* subsp. *paratuberculosis* with Inflammatory Bowel Disease

**DOI:** 10.1371/journal.pone.0148731

**Published:** 2016-02-05

**Authors:** Verlaine J. Timms, George Daskalopoulos, Hazel M. Mitchell, Brett A. Neilan

**Affiliations:** 1 School of Biotechnology and Biomolecular Sciences, Level 3, Biosciences Building, University of New South Wales, Sydney, Australia; 2 Inner West Endoscopy Centre, Endoscopy Services Pty. Ltd., Marrickville, Sydney, Australia; Universita di Sassari, ITALY

## Abstract

The association of *Mycobacterium avium* subspecies *paratuberculosis* (*M*. *paratuberculosis*) with Crohn’s disease is a controversial issue. *M*. *paratuberculosis* is detected by amplifying the *IS900* gene, as microbial culture is unreliable from humans. We determined the presence of *M*. *paratuberculosis* in patients with Crohn’s disease (CD) (n = 22), ulcerative colitis (UC) (n = 20), aphthous ulcers (n = 21) and controls (n = 42) using PCR assays validated on bovine tissue. Culture from human tissue was also performed. *M*. *paratuberculosis* prevalence in the CD and UC groups was compared to the prevalence in age and sex matched non-inflammatory bowel disease controls. Patients and controls were determined to be *M*. *paratuberculosis* positive if all three PCR assays were positive. A significant association was found between *M*. *paratuberculosis* and Crohn’s disease (p = 0.02) that was not related to age, gender, place of birth, smoking or alcohol intake. No significant association was detected between *M*. *paratuberculosis* and UC or aphthous ulcers; however, one *M*. *paratuberculosis* isolate was successfully cultured from a patient with UC. We report the resistance of this isolate to ethambutol, rifampin, clofazamine and streptomycin. Interestingly this isolate could not only survive but could grow slowly at 5°C. We demonstrate a significant association between *M*. *paratuberculosis* and CD using multiple pre-validated PCR assays and that *M*. *paratuberculosis* can be isolated from patients with UC.

## Introduction

Inflammatory Bowel Disease (IBD) and its divisions of Crohn’s disease (CD) and ulcerative colitis (UC) often strike in the prime of life and remain a life-long burden [1]. While the aetiology of IBD remains unclear, there is strong evidence to support the role of both microorganisms [2] and host genetic factors [3–6]. A number of microorganisms, including *Mycobacterium avium* subsp. *paratuberculosis (M*. *paratuberculosis)*, have been associated with IBD, but as yet, evidence to support the role of a specific microorganism in IBD is missing.

There has been considerable controversy regarding the potential role of *M*. *paratuberculosis* in CD. The prevalence of *M*. *paratuberculosis* in patients with CD and UC has been shown to be highly variable (92% and 0–35%, respectively) [7–9], however, a meta-analysis in 2007 [10] demonstrated a significant association between *M*. *paratuberculosis* and CD. A comparison of *M*. *paratuberculosis* prevalence in patients with CD, UC and controls was undertaken and in addition, samples from patients with aphthous ulcers of the GI tract (as opposed to oral aphthous ulcers) were included as it is currently believed that these are likely precursors of CD [11].

*M*. *paratuberculosis* can infect the gastrointestinal tract of a range of hosts and is the known cause of Johne’s disease in ruminants, a disease typified by diarrhoea, weight loss and eventual death [12]. The controversy regarding the association of *M*. *paratuberculosis* with CD relates in part to the fastidious nature of *M*. *paratuberculosis* and the consequent inability to reliably culture this organism. *M*. *paratuberculosis* is a subspecies of the *Mycobacterium avium* complex (MAC) and the genetic similarity between the subspecies of the MAC is greater than 97% [13, 14]. Given the high genetic similarity between MAC members, unique markers for the detection of *M*. *paratuberculosis* are limited. The majority of studies investigating the association of *M*. *paratuberculosis* and CD have used one marker, *IS900*, a unique insertion sequence of *M*. *paratuberculosis* [15–17].

Five PCR assays were applied to our patient set, all of which were previously validated on bovine tissue [18]. In addition, IBD patients were age and sex matched to non-IBD controls. Data on place of birth, smoking and other clinical data was collected on both patients and controls and collated with *M*. *paratuberculosis* status. A pure isolate of *M*. *paratuberculosis* was obtained from a patient with UC and a comprehensive biochemical and molecular characterisation of the second subculture of this isolate is also reported.

## Methods

### Ethics Statement

The study was approved by the Research Ethics Committee of the University of New South Wales (HREC 06233)/ (SESAHS (ES) 06/164). Written informed consent was obtained from study participants on forms approved by the above committee.

### Patients and samples

Biopsies were obtained from a total of 105 patients (including 42 controls), undergoing colonoscopy at the Inner West Endoscopy Centre, Marrickville, Sydney between January 2007 to December 2009. Controls, termed nIBD controls, were selected from patients undergoing colonoscopy for conditions unrelated to inflammatory bowel disease. Patients were included as controls if IBD was excluded on clinical and histopathological examination. One control was age (±5 years) and sex matched to each CD and UC patient for which PCR results were obtained. At the time of routine colonoscopy, mucosal biopsy specimens (approximately 20 mg wet weight) were collected from the terminal ileum or colon of each patient and control and placed in a sterile reaction tube. Samples were frozen at -20°C until they were transported to the laboratory (between 1–2 days) then stored at -80°C until required.

### Culture conditions and biochemical tests

Each biopsy was decontaminated in 60 μL of 0.75% (w/v) hexadecylpyridinium chloride (HPC) (Sigma) for 24 hours at room temperature. The HPC was then removed with a pipette and the biopsy washed in sterile H_2_0, then crushed between two sterile glass slides. To encourage growth from potential, as yet unknown growth factors that may be present in viable cultures of *M*. *paratuberculosis*, a sterile supernatant of a growing culture of *M*. *paratuberculosis* ATCC19698 was obtained using methodology outlined previously [19]. One hundred microlitres of this sterile supernatant was added to the crushed biopsies and inoculated onto Middlebrook 7H10 agar with 10% v/v oleic acid, albumin, dextrose and catalase supplement (Sigma) and 2 μg mycobactin J mL^-1^ (Allied Monitor). Two slopes, to which sterile supernatant only was added, were set up as controls with each batch. The sterile supernatant was prepared freshly each time and was never stored. The slopes were then incubated at 37°C, aerated weekly and checked for growth monthly for 18 months.

Standard Ziehl-Neelsen (Z-N) staining was performed on any apparent growth [20]. Growth on Lowenstein–Jensen (L-J) (Difco) with 2 μg mycobactin J mL^-1^, MacConkey (Oxoid) and mycobactin free L-J and Middlebrook 7H10 agar was assessed by adding a 10 μL inoculum of a McFarland no. 1 standard to the respective media and checking for growth over 3 months. Thermostable and semiquantitative standard catalase assays were performed [20]. Bacterial size was determined using the AxioVision LE software.

To confirm the identity of the new isolate, strain 43525, DNA extraction for PCR assays and biochemical tests were conducted on the second subculture as there was insufficient growth on the first subculture. A previously published PCR assay was used to determine whether strain 43525 was a Cow (C) or a Sheep (S) strain [21]. Automated sequencing to identify PCR products was carried out using the PRISM BigDyeTM cycle sequencing system v3.1 and ABI 3730 capillary Applied Biosystem.

### DNA extraction and PCR assays

DNA was extracted from biopsies using a method from previously published studies [15, 16, 18]. The primers used for the nested *IS900* assay and the single round *IS900*, f57, myco16S and universal 16S assays were validated in previous publications [15, 22–25]. The conditions for each PCR assay were validated on bovine tissue in a previous study [18] and the sensitivity of the *IS900*, nested *IS900* and myco16S PCR assays was calculated to be 80% while the sensitivity of the f57 assay was 60%. The single round *IS900* PCR assay was performed using 5 mM MgCl_2_, 100 μM dNTPs, 1 x reaction buffer, 1 U *Taq*, 2.5 μM of each primer and 5 μL (corresponding to 20–100 ng) of DNA sample per tube. Cycle conditions were as follows: 96°C for 1 min, then 96°C for 15 s, 50°C for 15 s, 72°C for 1 min for 35 cycles then 72°C for 5 min and 20°C hold.

For the nested *IS900* PCR, the conditions were for the first stage; 1.25 mM MgCl_2,_ 50 μM of dNTPs, 1 x reaction buffer, 1 U of *Taq*, 2 μM each primer and 2 μL (corresponding to 20–100 ng) of DNA sample added together and made up to 20 μL per tube. Cycling conditions were: 94°C for 1 min, 94°C for 10 s, 50°C annealing for 20 s, 72°C extension for 30 s for 35 cycles, then 72°C for 7 min. For the second stage 1.25 mM MgCl_2,_ 100 μM of dNTPs, 1 x reaction buffer, 0.4 U *Taq*, 5 μM of primers and 5 μL of PCR product from the first stage was added together and made up to 20 μL per tube. The reaction conditions were as follows: 94°C for 1 min, then 94°C for 10 s, 58°C for 20 s, 72°C for 30 s for 30 cycles followed by 72°C for 7 min.

For the f57 PCR assay 0.75 mM MgCl_2,_ 50 μM dNTPs, 1 x reaction buffer, 0.5 U Taq, 10 μM primer and 5 μL (corresponding to 20–100 ng) DNA were added together and made up to 20 μL. The cycling conditions were: 95°C for 4 mins, 94°C for 45 s, 62°C for 45 s, 72°C for 45 s for 40 cycles then 72°C 10 min and hold at 20°C.

The myco16S PCR was performed using 1.25 mM MgCl_2,_ 100 μM dNTPs, 1 x reaction buffer, 0.4 U *Taq*, 5 μM of each primer and 2 μL (corresponding to 20–100 ng) DNA added together and made up to 20 μL. The cycling conditions were: 95°C for 3 mins, then 94°C for 30 s, 56°C for 1 min, 72°C for 1 min for 30 cycles and then 72°C 2 min.

The 16S rRNA gene PCR assay was performed using the following conditions: 2.5 mM MgCl_2_, 200 μM dNTPs, 1 x reaction buffer, 1 U of *Taq* polymerase, 0.4 μM of 16S rRNA gene primers [25] and 2 μL of DNA added together, and made up to 20 μL with sterilised distilled water. The PCR conditions were the following: 95°C for 3 min, then 94°C 30 s, 56°C 1 min, 72°C 1 min for 30 cycles then 7 min at 72°C. Patients were classified as *M*. *paratuberculosis* positive if all three PCR assays (*IS900*, f57 and nested *IS900*) were positive.

### Growth at 5°C

Isolate 43525 appeared to be growing on a slope placed in a refrigerator, therefore we determined how fast isolate 43525 could grow at 5°C. A 20 μL bacterial suspension at a concentration equal to a McFarland No. 1 standard was added to Middlebrook 7H9 broth containing 10% albumin, dextrose and catalase (ADC) (Difco) and 2 μg mycobactin J mL^-1^. Flasks were incubated at 5°C with shaking and each week, for 6 weeks a 2 μL aliquot was plated onto slopes of Middlebrook 7H10 agar with 2 μg mycobactin J mL^-1^ to determine Colony Forming Units (CFU).

### Antibiotic susceptibility

Antibiotic susceptibility and resistance of isolate 43525 was determined in triplicate by the agar proportion method as previously described [26]. Clarithromycin, rifampin and clofazimine were tested at 1–4 μg mL^-1^ while ciprofloxacin, ethambutol and streptomycin were tested in the range of 2–8 μg mL^-1^, all using Middlebrook 7H10 agar with 2 μg mycobactin J mL^-1^. An inoculum equivalent to a McFarland No.1 standard was added to slopes and incubated for 3 weeks. Any negative slopes were left for a total of 12 weeks.

### Statistical analysis

The χ^2^ test and Kruskal-Wallis test was used to analyse the effect of factors such as age, gender, smoking, alcohol and place of birth. The Fisher’s exact test (two tailed) was used to compare the prevalence of *M*. *paratuberculosis* in patients and controls (Graphpad Prism software).

## Results

Of the 105 patients in the study, 22 patients had CD (13 male, 59%), 20 had UC (8 male, 40%), 21 had aphthous ulcers of the terminal ileum (13 male, 62%) and 42 were non-IBD (nIBD) controls. Patient characteristics including place of birth and smoking are presented in Table 1. Of the CD patients that were *M*. *paratuberculosis* positive (n = 6), four had CD of the terminal ileum (L1) and two had CD of the ileum and colon (L3). Of the UC patients that were *M*. *paratuberculosis* positive (n = 3), two had left-sided disease (E2) and one had extensive disease (E3), according to the Montreal classification [27]. The results of the PCR assays are presented in Table 2. The three PCR assays used to detect *M*. *paratuberculosis* across patients and controls gave variable results (Table 3). As observed in Table 3, in the CD control group not one patient was positive for *M*. *paratuberculosis* across the three assays and only one patient was positive in two assays (*IS900* and f57). Conversely, in the CD group, six patients were *M*. *paratuberculosis* positive across all three assays.

**Table 1:** Characteristics of patients included in the study.

		CD	UC	Aphthous Ulcers	Controls
**Number**		22 (13 male)	20 (8 male)	21 (13 male)	42 (21 male)
**Median age (range)**		35 (20–86)	36 (16–80)	54 (20–71)	38 (18–83)
**Place of birth**	**Australia**	10	9	9	16
	**Europe**	6	8	5	24
	**Middle East**	3	2	5	0
	**Asia**	2	1	1	1
	**Other**	1	0	1	1
**Smoking (cig/day)**	**0**	19	20	13	34
	**>5**	2	0	3	4
	**>20**	1	0	5	4
**Alcohol (g/wk)**	**0**	17	14	15	24
	**>10**	1	1	1	5
	**>20**	4	5	5	13
**Initial presentation**	**Yes**	16	15	13	n/a
	**No**	6	5	8	
**Previous IBD Treatment** (If not initial presentation)		2	2	1	n/a*

* One control patient had taken flagyl for diarrhoea that was not associated with IBD.

**Table 2:** The *M*. *paratuberculosis* (MAP) prevalence in patients with either Crohn’s or UC as compared to age and sex-matched controls.

		MAP positive	MAP negative
**CD group**	Patients (n = 21)	6 (29%)	15 (71%)
	Controls (n = 21)	0	21 (100%)
**UC group**	Patients (n = 17)	3 (18%)	14 (82%)
	Controls (n = 17)	2 (12%)	15 (88%)

**Table 3:** A comparison of each individual PCR assay and the patient/ control samples (by identity number) that were *M*. *paratuberculosis* positive.

PCR assay	CD patients	CD CONTROLS	UC patients	UC CONTROLS
	MAP positive sample number	MAP positive sample number	MAP positive sample number	MAP positive sample number
**n*IS900***	**CD2**,**CD4,CD5,CD11,CD18,CD21**	nIBD6,nIBD30	**UC26,UC27,UC32**	**nIBD14,nIBD34**
***IS900***	**CD2**,CD3,**CD4,CD5**,CD6,CD10,**CD11**,CD12,CD14,CD17,**CD18,CD21**	nIBD3,nIBD4,nIBD7,nIBD10,nIBD12,nIBD36	UC25,**UC26,UC27**,UC28,**UC32**,UC33,UC38	nIBD9,**nIBD14,nIBD34**
**f57**	**CD2**,**CD4,CD5**,CD6,CD8,**11,18,21**	nIBD11,nIBD12,nIBD13	UC25,**UC26,UC27**,UC28,**UC32**	**nIBD14,nIBD34**

Each patient sample from each group (ie CD, UC and “C” for controls) is represented by a number. The samples that were positive in all three assays are in bold.

In 12 patients, the DNA sample produced no PCR product with any of the assays. These samples were obtained from, one patient with CD, three with UC, eight with aphthous ulcers. For the aphthous ulcers group, 2/21 patients were positive with the single round *IS900* only and no other PCR assay, therefore this group was not compared to a control group.

Four patients that were not *M*. *paratuberculosis* positive were found to be positive by the myco16S PCR. In two samples, the product quality was too poor to be sequenced. In the other two patients (one with UC and one control) sequencing found that the 16S rRNA gene product matched *M*. *abscessus* subsp. *bolleti* or *M*. *massiliense*. These two mycobacterial species have identical 16S rRNA genes and therefore cannot be distinguished by the sequence of this gene.

No significant difference was observed between the CD, UC or aphthous ulcer group and controls in regard to age, place of birth, smoking or alcohol intake (Table 1). A significant association was found between *M*. *paratuberculosis* PCR positivity and CD (p = 0.02) (Table 2).

For culture, isolate 43525, was obtained from the colon of a 66 year old female with UC. The patient was Australian born, did not smoke or drink alcohol and was suffering diarrhoea. Histological examination of the colonic mucosa revealed diffuse inflammation of both an acute and chronic nature consistent with UC. Although this was her first presentation, she underwent a total colectomy six months after the biopsy sample was taken, in which histological examination of the resected colon remained consistent with the diagnosis of active chronic UC.

Isolate 43525 initially took 40 weeks to grow at 37°C (S1A Fig), however, following the first subculture it grew vigorously on solid media. Attempts were made to amplify the *IS900* and f57 genes directly on another colonic biopsy from the same patient without success. In addition, a universal 16S rRNA gene PCR assay also failed to produce a band. Unfortunately, this was all the material we had from that patient. The major characteristics of this isolate are outlined in Table 4 and are compared to the type strain ATCC19698 and the biochemical properties of human strains characterised in a previous publication [28].

**Table 4:** Comparison of the biochemical test results for isolate 43525 compared to other *M*. *paratuberculosis* isolates reported in the literature.

Test	ATCC19698	43525	Chiodini strains^
Colony morphology	smooth	rough	rough
**Size (μm)**	1.5–1.6 x 0.7	1–1.5 x 0.4	1.8–2.3 x 0.3
**Growth Rate 1**^**o**^ **culture**	NA	40 weeks	15–72 weeks
**Mycobactin dependent**	yes	yes*	yes
**Growth at 5**^**°**^**C**	no	slow#	unknown
**Growth at 44**^**°**^**C**	+	+	none (42°C)
**L-J +** 2 μg/mL mycobactin J	+	+	-
**Catalase (Room Temp)**	<45 mm	<45 mm	<45 mm
**Thermostable catalase**	+	+	+
***IS900***	+	+	+
**f57**	+	+	+

^ [28]

* The mycobactin dependency of 43525 was apparent on L-J media only. When tested on Middlebrook 7H10 agar without mycobactin the strain was independent.

# See Fig 1

### Growth at 5°C

**Fig 1:**
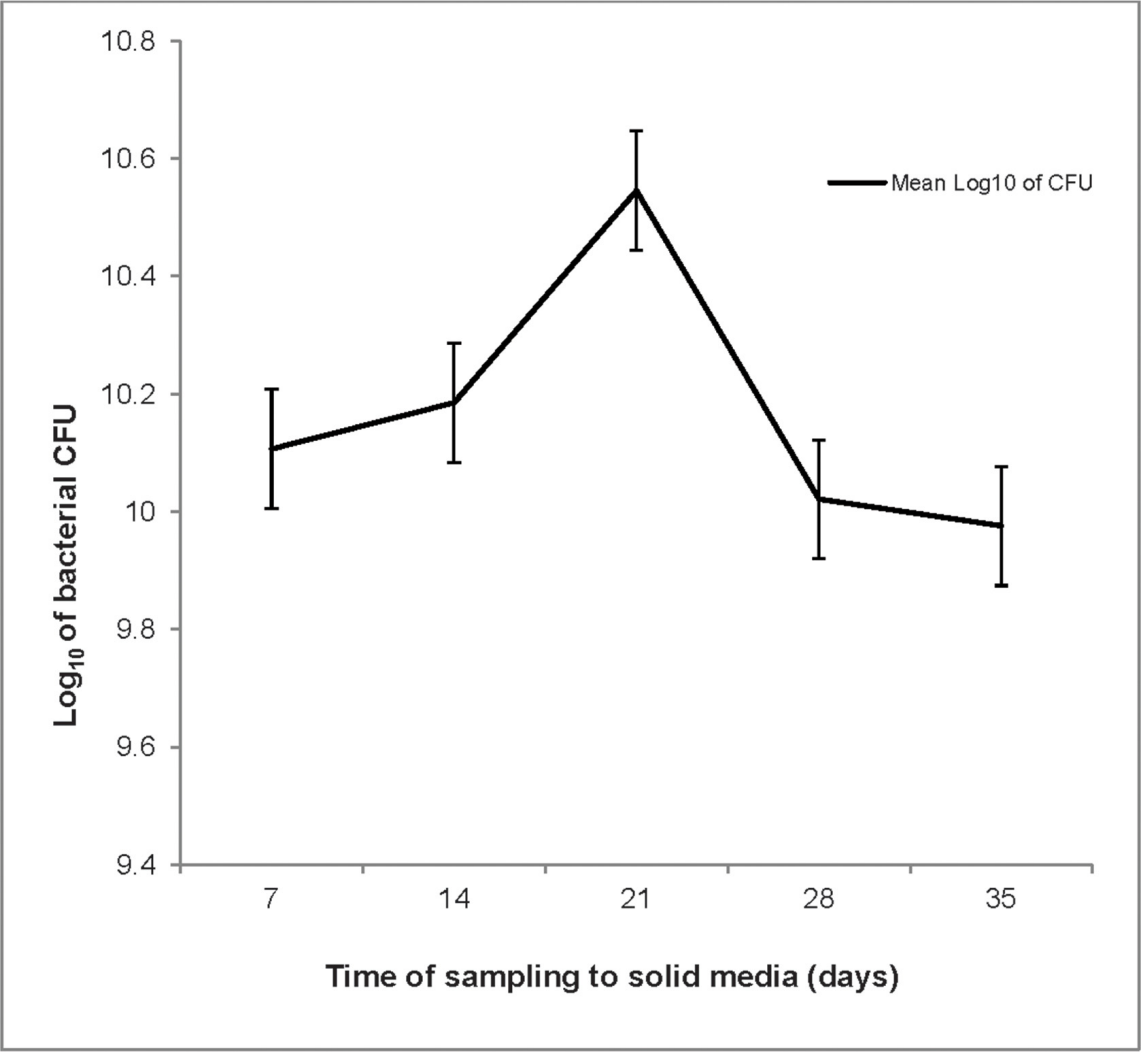
Natural log of Colony Forming Units (CFU) of isolate 43525 growing at 5°C. Error bars indicate standard deviation. The increase in CFUs on day 21 was greater than 2 standard deviations.

Isolate 43525 grew at 5°C, as determined by the viable count method. The highest cell count was retrieved three weeks after inoculation (Fig 1). This experiment was repeated twice, with duplicate cultures and a negative control included each time, with the same results obtained. The temperature fluctuations of the cold incubator were recorded between 5.02–4.52°C, with a mean temperature of 4.62°C.

### Mycobactin independence

Isolate 43525 was C type and when grown on Middlebrook 7H10 agar without mycobactin, a perceptible reduction in the number of colonies, as compared with media containing mycobactin, was observed (S1B and S1C Fig). To confirm this, colonies were picked from media with and without mycobactin and streaked on to mycobactin-free Middlebrook 7H10. All colonies retained the ability to grow on mycobactin-free Middlebrook 7H10. In contrast, no growth was ever obtained on mycobactin-free L-J media, however growth was apparent when mycobactin was added. The mycobactin dependency was only apparent on L-J media. The sequences for *IS900* and f57 of strain 43525 were 100% identical to those of *M*. *paratuberculosis* strains ATCC19698 and K10 (Table 4).

### Antibiotic susceptibility

The MICs for ciprofloxacin, streptomycin, clofazimine and rifampin were higher for 43525 as compared with ATCC19698 (Table 5), while the MIC of clarithromycin was lower.

**Table 5:** Comparison of MIC values between ATCC19698 and isolate 43525.

Antibiotic Tested	ATCC19698 MIC (μg mL^-1^)	43525 MIC (μg mL^-1^)
**Ciprofloxacin**	<2	8
**Clarithromycin**	>4	<1
**Ethambutol**	>8	>8
**Streptomycin**	8	>8
**Clofazamine**	2	>4
**Rifampin**	4	>4

## Discussion

The current study is the first to employ and to compare the detection rates of *M*. *paratuberculosis* in human tissue using two *IS900* assays and the f57 assay. As *M*. *paratuberculosis* culture from humans is unreliable and there are a limited number of unique markers, a sample was deemed positive for *M*. *paratuberculosis* only if all three assays were positive. Using these criteria, we found the prevalence of *M*. *paratuberculosis* to be significantly higher (p = 0.02) in CD patients (29%) as compared with controls (0%). In contrast, no significant difference was found with the prevalence of *M*. *paratuberculosis* in patients with UC or aphthous ulcers.

We showed that the nested *IS900* assay and f57 assay produced comparable results, while the single round *IS900* assay resulted in a higher percentage of positive samples across all groups. These results are in agreement with the reported heterogeneity of *IS900* detection, that is, the lack of specificity of the *IS900* assay, in human tissue and the recommendation that the diagnosis of *M*. *paratuberculosis* should not rely upon a single *IS900* result [7, 29].

Although the lack of concordance in the *M*. *paratuberculosis* positivity across assays can be explained in some part by the lack of specificity of the *IS900* it cannot totally explain this phenomenon. It is possible that these results could reflect the presence of f57 and *IS900*-like sequences in the human gut microbiome in this group of subjects, particularly since only 20% of the genetic composition of the gut microbiome is known [30]. A follow-up of patients such as these may shed light on whether these PCR results are wholly due to the lack of specificity of the PCR assay or are an indication of transient bacteria passing through the human gastrointestinal tract. In addition, an internal positive control (IPC) was used to identify samples containing PCR inhibitors. The 16S rRNA PCR assay does not impair detection sensitivity by competing with the target DNA for reaction components. However, in light of the lack of concordance across in the *M*. *paratuberculosis* positivity across assays, the inclusion of a defined exogenous IPC such as that used in previous studies [15] could provide additional insight and may lead to more reliable detection.

Although it has been suggested that aphthous ulcers of the GI tract are precursors of CD, we found no evidence of *M*. *paratuberculosis* infection in any of our patients with aphthous ulcers. If aphthous ulcers are indeed a precursor of CD our results raise the question as to when *M*. *paratuberculosis* infection may occur. One possible scenario is that *M*. *paratuberculosis* may colonise only once inflammation has been initiated.

To our knowledge, this is also the only study to have matched controls by age and sex when investigating the association of *M*. *paratuberculosis* with CD. Although not reflected in our patient set, the majority of CD cases are usually women, in contrast to UC where both sexes are equally affected [31]. Significant differences in age and sex between CD, UC and controls have been demonstrated previously in studies exploring *M*. *paratuberculosis* associated CD, hence we endeavoured to remove these possibly confounding factors [15, 16].

Having established that *M*. *paratuberculosis* can be isolated from the human GI tract in this study [28, 32, 33] and the repeated demonstration of the association of *M*. *paratuberculosis* with some CD cases [10], future studies could investigate whether treating patients with *M*. *paratuberculosis* infection would benefit their disease course. In 2007, the Australian IBD study endeavoured to treat *M*. *paratuberculosis* associated CD, using a combination antibiotic therapy (clarithromycin, rifabutin and clofazamine) for up to 2 years [34]. A sustained benefit was not reported in the Australian study, however, the treatment administered in that study and a clinical trial currently recruiting [35], is based on limited knowledge as to what constitutes a successful *M*. *paratuberculosis* antibiotic treatment regimen in humans. The antibiotic profile of human isolate 43525 demonstrated differences in the susceptibility pattern as compared with bovine and other human *M*. *paratuberculosis* isolates [28]. In line with a previous study, isolate 43525 was resistant to ethambutol, clofazimine and rifampin [36]. Whether this resistance is widespread among *M*. *paratuberculosis* remains to be investigated. Based on the antibiotic resistance pattern reported here, use of the currently recommended antibiotic regimens would be ineffective, thus improvement in the symptomatology associated with CD would be unlikely.

Mycobactin dependency is still used to differentiate *M*. *paratuberculosis* from other subspecies of the *M*. *avium* complex. The mycobactin dependency of isolate 43525 was media based, hence, in a clinical laboratory this isolate may be misidentified as *M*. *avium* subsp. *avium*, an organism with different consequences in human infection. Interestingly, ovine isolates of *M*. *paratuberculosis* have also been found to grow on Middlebrook agar without the addition of mycobactin [37] and may be explained by the finding that the mycobactin operon promoter is active in *M*. *paratuberculosis* [38]. The genome of *M*. *paratuberculosis* 43525 has since been sequenced and the mycobactin cluster was found to differ to the mycobactin clusters of other *M*. *paratuberculosis* isolates [39]. In addition, isolate 43525 grew across a range of temperatures from 5°C to 44°C. *M*. *smegmatis* has been shown to grow at 10°C but was not tested below this [40]. The significance of this requires further investigation, particularly whether growth can also occur in milk, given reports that *M*. *paratuberculosis* survives pasteurisation [41].

The mycobacteria contain species that are ubiquitous in the environment and are some of the most persistent pathogens known to man. *M*. *paratuberculosis*, the little known subspecies of the MAC complex is a pathogen of animals and shares 97% of its genetic makeup with known human pathogens of that complex [42]. This is the first study to apply PCR assays to human tissue that have been pre-validated on a panel of *M*. *paratuberculosis* infected and non-infected bovine tissue [18]. In the absence of more reliable microbial culture data, combining three pre-validated PCR assays is an important step forward for confirming the detection of *M*. *paratuberculosis* in human mucosal biopsies. Like the study by Naser et. al. in 2004 [32], we report the characterisation of *M*. *paratuberculosis* from a patient with UC with a view that further work should strive to improve our technical ability to detect and monitor the presence of this species in humans.

## Supporting Information

S1 Fig Growth of 43525. A) The appearance of original slope of isolate 43525, 8 weeks after growth first appeared, B) Isolate 43525 growing on Middlebrook 7H10 without mycobactin, C) Isolate 43525 growing on Middlebrook 7H10 with mycobactin added.
(TIF)
