# Supplementary figures and images for: The Association of Mycobacterium avium subsp. paratuberculosis with Inflammatory Bowel Disease
Source: PLoS One. 2016 Feb 5;11(2):e0148731. doi: 10.1371/journal.pone.0148731 (PMC4746060; doi:10.1371/journal.pone.0148731)

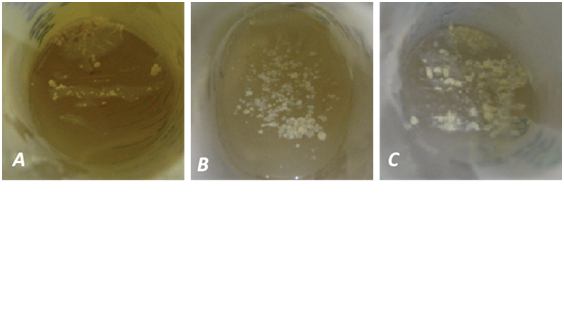

Supplement: S1 Fig — A) The appearance of original slope of isolate 43525, 8 weeks after growth first appeared, B) Isolate 43525 growing on Middlebrook 7H10 without mycobactin, C) Isolate 43525 growing on Middlebrook 7H10 with mycobactin added. (TIF) [file pone.0148731.s001.tif]
